# Crystal structure and Hirshfeld surface analysis of di­aqua­bis­(*N*,*N*-di­ethyl­nicotinamide-κ*N* ^1^)bis­(2,4,6-tri­methyl­benzoato-κ*O*)manganese(II)

**DOI:** 10.1107/S2056989018003377

**Published:** 2018-03-02

**Authors:** Tuncer Hökelek, Safiye Özkaya, Hacali Necefoğlu

**Affiliations:** a Department of Physics, Hacettepe University, 06800 Beytepe, Ankara, Turkey; b Department of Chemistry, Kafkas University, 36100 Kars, Turkey; c International Scientific Research Centre, Baku State University, 1148 Baku, Azerbaijan

**Keywords:** crystal structure, manganese(II), transition metal complexes of benzoic acid and nicotinamide derivatives

## Abstract

In the title centrosymmetric complex, [Mn(C_10_H_11_O_2_)_2_(C_10_H_14_N_2_O)_2_(H_2_O)_2_], the Mn^II^ cation is located on an inversion centre. The four O atoms form a slightly distorted square-planar arrangement around the Mn^II^ cation, and the distorted octa­hedral coordination is completed by two pyridine N atoms at distances of 2.3289 (15) Å. The dihedral angle between the planar carboxyl­ate group and the adjacent benzene ring is 87.73 (16)°, while the benzene and pyridine rings are oriented at a dihedral angle of 43.03 (8)°. In the crystal, the water mol­ecules are involved in both intra­molecular (to the non-coordinating carboxyl­ate O atom) and inter­molecular (to the amide carbonyl O atom) O—H⋯O hydrogen bonds. The latter lead to the formation of layers parallel to (100). These layers are further linked *via* weak C—H⋯O hydrogen bonds, resulting in a three-dimensional supra­molecular network. The Hirshfeld surface analysis of the crystal structure indicates that the most important contributions for the crystal packing are from H⋯H (70.0%), H⋯O/O⋯H (15.5%) and H⋯C/C⋯H (14.0%) inter­actions. One of the ethyl groups of the di­ethyl­nicotinamide ligand is disordered over two sets of sites, with an occupancy ratio of 0.282 (10):0.718 (10).

## Abstract 2

The Mn^II^ complex is centrosymmetric and the mol­ecules are linked by O—H⋯O and C—H⋯O hydrogen bonds into the three-dimensional supra­molecular network.

## Chemical context   

Nicotinamide (NA) is one form of niacin. A deficiency of this vitamin leads to loss of copper from the body, known as pellagra disease. Victims of pellagra show unusually high serum and urinary copper levels (Krishnamachari, 1974 ▸). The nicotinic acid derivative *N*,*N*-Di­ethyl­nicotinamide (DENA) is an important respiratory stimulant (Bigoli *et al.*, 1972 ▸). The crystal structure of the complex [Co(CH_3_CO_2_)_2_(DENA)_2_(H_2_O)_2_] (Mikelashvili, 1982 ▸) is isostructural with the analogous Ni, Mn, Zn and Cd complexes (Sergienko *et al.*, 1980 ▸). The structures of some complexes obtained from the reactions of transition metal(II) ions with nicotinamide (NA), *N*,*N*-Di­ethyl­nicotinamide (DENA) and some benzoic acid derivatives as ligands, *e.g*. [Zn(NA)_2_(C_7_H_5_O_3_)_2_] [(II); Necefoğlu *et al.*, 2002 ▸], [Zn(NA)_2_(C_8_H_8_NO_2_)_2_]·H_2_O [(III); Hökelek *et al.*, 2009*a* ▸], [Co(NA)(C_9_H_10_NO_2_)_2_(H_2_O)_2_] [(IV); Hökelek *et al.*, 2009*b* ▸], [Zn_2_(DENA)_2_(C_11_H_14_NO_2_)_4_] [(V); Hökelek *et al.*, 2009*c* ▸], [Mn(DENA)_2_(C_7_H_4_ClO_2_)_4_(H_2_O)_2_ [(VI); Hökelek *et al.*, 2009*d* ▸], [Mn(DENA)_2_(NCS)_2_] [(VII); Bigoli *et al.*, 1973*a* ▸], [Zn(DENA)_2_(NCS)_2_(H_2_O)_2_] [(VIII); Bigoli *et al.*, 1973*b* ▸] and [Cd(DENA)(SCN)_2_] [(IX); Bigoli *et al.*, 1972 ▸] have been determined previously. In complex (VII), DENA is a bidentate ligand, while in complexes (V), (VI), (VIII) and (IX), DENA is a monodentate ligand. In complex (V), the four 4-(di­ethyl­amino)­benzoate (DEAB) ions act as bidentate ligands bridging the two Zn atoms.
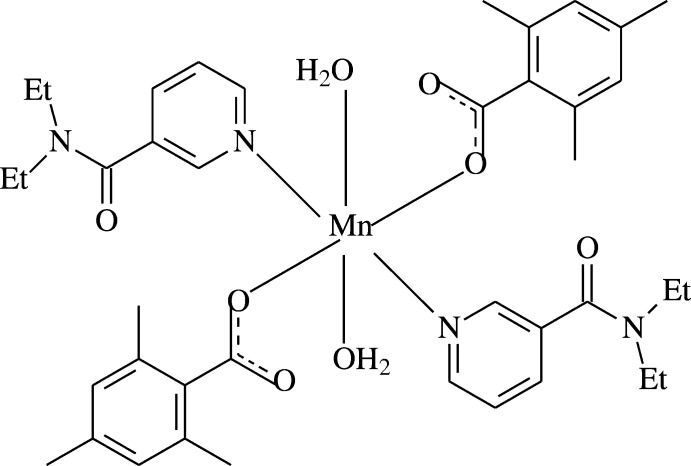


The structure–function–coordination relationships of the aryl­carboxyl­ate ion in Mn^II^ complexes of benzoic acid deriv­atives may change depending on the nature and position of the substituted groups on the benzene ring, the nature of the additional ligand mol­ecule or solvent, and the pH and temperature of synthesis (Shnulin *et al.*, 1981 ▸; Nadzhafov *et al.*, 1981 ▸; Antsyshkina *et al.*, 1980 ▸; Adiwidjaja *et al.*, 1978 ▸). When pyridine and its derivatives are used instead of water mol­ecules, the structure is completely different (Catterick *et al.*, 1974 ▸). In this context, the Mn^II^-containing title compound, (I), with 2,4,6-tri­methyl­benzoate (TMB) and DENA ligands, namely di­aqua­bis­(*N*,*N*-di­ethyl­nicotinamide -*κN* ^1^)bis­(2,4,6-tri­methyl­benzoato-*κO* ^1^) manganese(II), [Mn(DENA)_2_(TMB)_2_(H_2_O)_2_], was synthesized and its crystal structure is reported on herein.

## Structural commentary   

**Figure 1:**
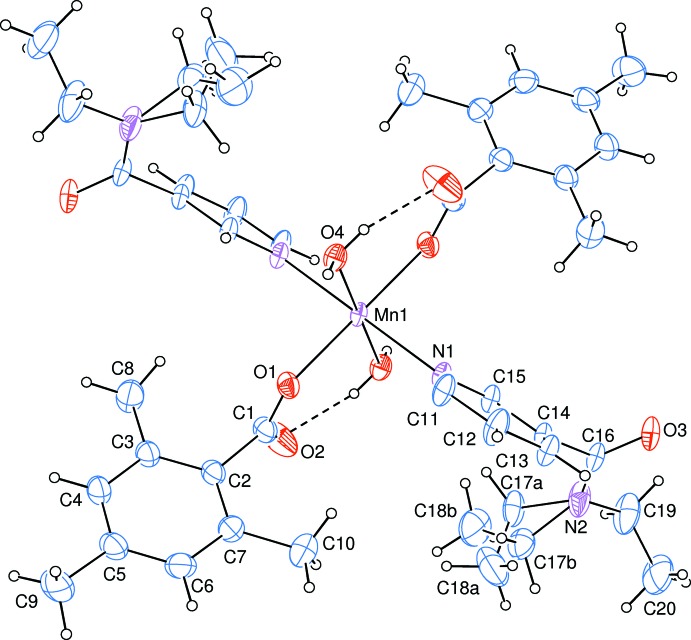
The mol­ecular structure of the title complex with the atom-numbering scheme for the asymmetric unit. Unlabelled atoms are related to labelled ones by the symmetry operation (−*x*, −*y*, −*z*). Displacement ellipsoids are drawn at the 50% probability level. Intra­molecular O—H⋯O hydrogen bonds, enclosing *S*(6) ring motifs, are shown as dashed lines.

The asymmetric unit of the crystal structure of the mononuclear title complex, contains one Mn^II^ cation located on an inversion centre, one 2,4,6-tri­methyl­benzoate (TMB) anion and one *N*,*N*-di­ethyl­nicotinamide (DENA) mol­ecule together with the one water mol­ecule, with all ligands coordinating to the Mn^II^ cation in a monodentate manner (Fig. 1 ▸).

The Mn^II^ cation is coordinated monodentately through the two carboxyl­ate O atoms (O1 and O1^i^) of the two symmetry-related TMB anions and the two symmetry-related water O atoms (O4 and O4^i^) at distances of 2.0999 (14) and 2.2230 (15) Å, respectively, to form a slightly distorted square-planar arrangement, while the slightly distorted octa­hedral coordination sphere is completed by the two pyridine N atoms (N1 and N1^i^) at distances of 2.3289 (15) Å of the two symmetry-related DENA ligands in the axial positions [symmetry code: (i) −*x*, −*y*, −*z*] (Fig. 1 ▸).

The near equalities of the C1—O1 [1.254 (3) Å] and C1—O2 [1.243 (3) Å] bonds in the carboxyl­ate groups indicate delocalized bonding arrangements, rather than localized single and double bonds. The Mn—O bond lengths [2.2230 (15) Å] for water oxygen atoms are by *ca* 0.1 Å longer than those involving the benzoate oxygen atoms [2.0999 (14) Å]. The Mn—N bond length [2.3289 (15) Å] is the longest one in the MnO_4_N_2_ octa­hedron. The Mn1 atom lies 0.0697 (1) Å above the planar (O1/O2/C1) carboxyl­ate group. The O2—C1—O1 bond angle [125.5 (2)°] seems to be significantly increased than that present in a free acid [122.2°], in which the O2—C1—O1 bond angle may be compared with the corresponding values of 123.5 (2) and 120.4 (2)° in (II), 119.2 (3) and 123.8 (2)° in (III), 123.86 (13) and 118.49 (14)° in (IV), 125.11 (13) and 124.80 (14)° in (V) and 126.65 (14)° in (VI), where the benzoate ions are coordinated to the metal atoms only bidentately in (V), only monodentately in (VI) and both monodentately and bidentately in (II), (III) and (IV). The O—Mn—O and O–Mn—N bond angles [range 87.88 (6) to 92.12 (6)° for *cis* angles; all *trans* angles are 180° due to symmetry] deviate slightly from ideal values, with same average values of 90.00 (6)°.

The dihedral angle between the planar carboxyl­ate group (O1/O2/C1) and the adjacent benzene *A* (C2–C7) ring is 87.73 (16)°, while the benzene *A* and pyridine *B* (N1/C11–C15) rings are oriented at a dihedral angle of *A*/*B* = 43.03 (8)°.

## Supra­molecular features   

**Figure 2:**
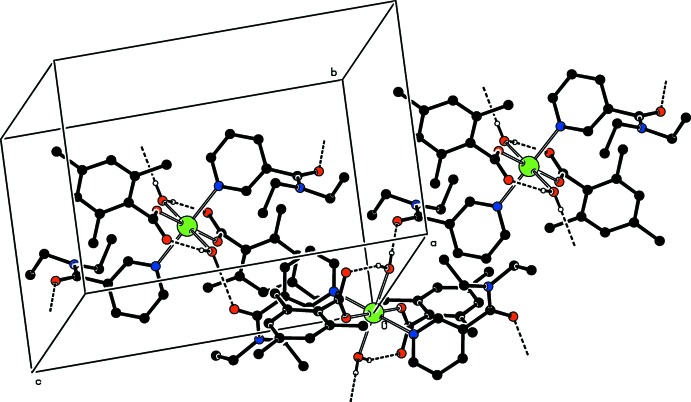
Part of the crystal structure. Only O—H_W_⋯O_TMB_ and O—H_W_⋯O_DENA_ (W = water, TMB = 2,4,6-tri­methyl­benzoate and DENA = *N*,*N*-di­ethyl­nicotinamide) hydrogen bonds, enclosing *S*(6) ring motifs, are shown as dashed lines. Only one part of the disordered group has been included and the C-bound hydrogen atoms have been omitted for clarity.

Intra­molecular O—H_w_⋯O_c_ (w = water, c = non-coordinating carboxyl­ate O atom) hydrogen bonds (Table 1 ▸) link two of the water ligands to the TMB anions, enclosing an *S*(6) ring motif (Fig. 1 ▸). The other water H atom is involved in inter­molecular O—H_w_⋯O_DENA_ (O_DENA_ = carbonyl O atom of *N*,*N*-di­ethyl­nicotinamide) hydrogen bonds (Table 1 ▸), leading to the formation of layers parallel to (100) (Fig. 2 ▸). These layers are further linked into a three-dimensional network structure *via* weak C—H_TMB_⋯O_c_ (TMB = 2,4,6-tri­methyl­benzoate) and C—H_DENA_⋯O_DENA_ hdyrogen bonds (Table 1 ▸).

## Hirshfeld surface analysis   

**Figure 3:**
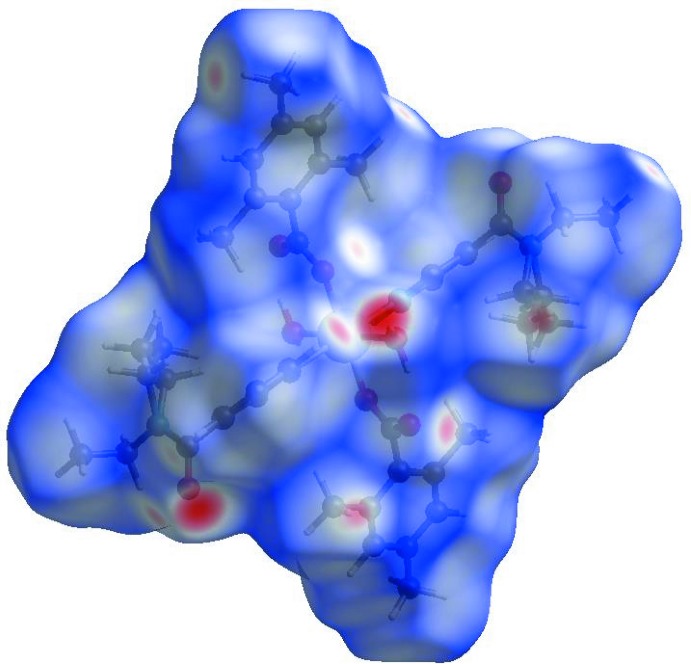
View of the three-dimensional Hirshfeld surface of the title complex plotted over *d*_norm_ in the range −0.6741 to 1.6440 a.u.

**Figure 4:**
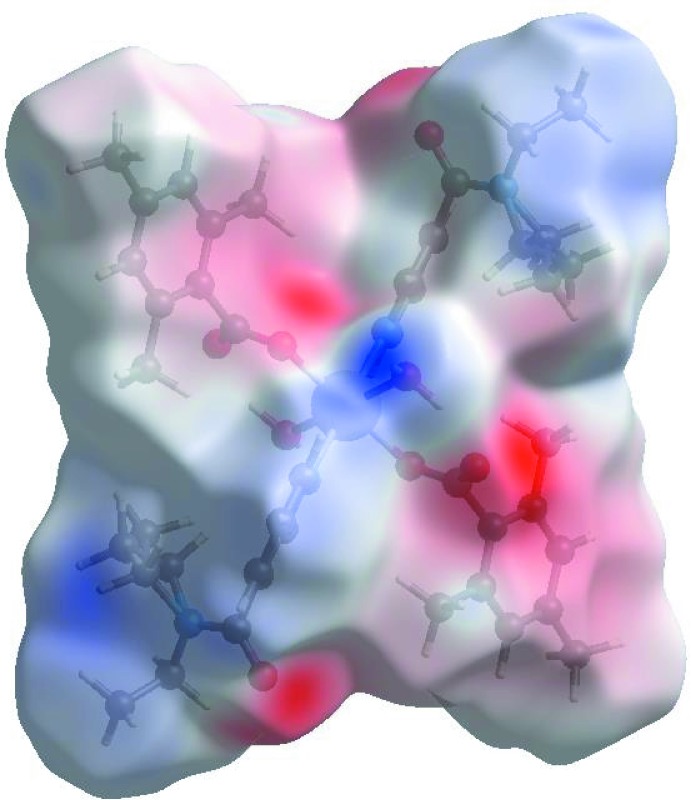
View of the three-dimensional Hirshfeld surface of the title complex plotted over electrostatic potential energy in the range −0.1032 to 0.1415 a.u. using the STO-3G basis set at the Hartree–Fock level of theory. The O—H⋯O and C—H⋯O hydrogen-bond donors and acceptors are viewed as blue and red regions around the atoms corresponding to positive and negative potentials, respectively.

**Figure 5:**
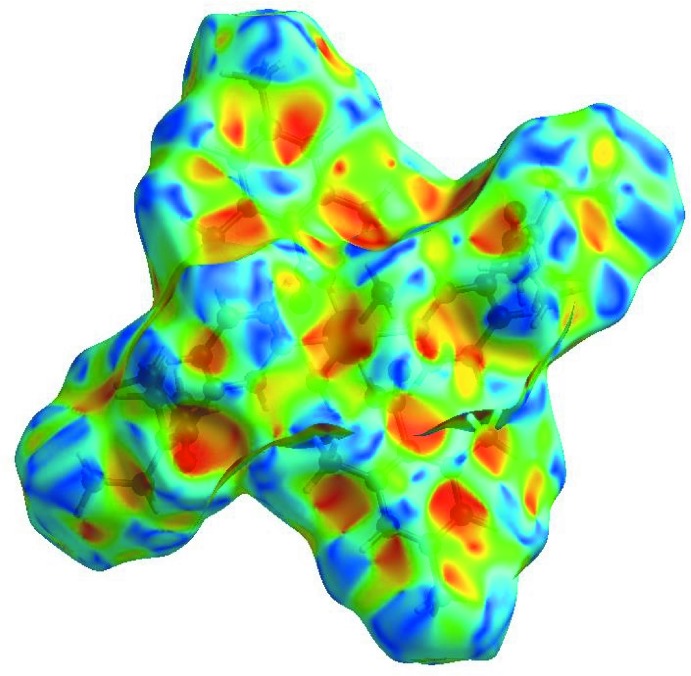
Hirshfeld surface of the title complex plotted over shape-index.

Visulization and exploration of inter­molecular close contacts in the crystal structure of the title complex is invaluable. Thus, a Hirshfeld surface (HS) analysis (Hirshfeld, 1977 ▸; Spackman & Jayatilaka, 2009 ▸) was carried out by using CrystalExplorer*17.5* (Turner *et al.*, 2017 ▸) to investigate the locations of atom–atom short contacts with potential to form hydrogen bonds and the qu­anti­tative ratios of these inter­actions and those of the π-stacking inter­actions. In the HS plotted over *d*_norm_ (Fig. 3 ▸), the white surface indicates contacts with distances equal to the sum of van der Waals radii, and the red and blue colours indicate distances shorter (in close contact) or longer (distinct contact) than the van der Waals radii, respectively (Venkatesan *et al.*, 2016 ▸). The bright-red spots appearing near DENA-O3, carboxyl­ate-O2, and hydrogen atoms H41, H42, H9*C* and H11 indicate their roles as the respective donors and acceptors in the dominant O—H⋯O and C—H⋯O hydrogen bonds; they also appear as blue and red regions corresponding to positive and negative potentials on the HS mapped over electrostatic potential (Spackman *et al.*, 2008 ▸; Jayatilaka *et al.*, 2005 ▸) as shown in Fig. 4 ▸. The blue regions indicate the positive electrostatic potential (hydrogen-bond donors), while the red regions indicate the negative electrostatic potential (hydrogen-bond acceptors). The shape-index of the HS is a tool to visualize the π–π stacking inter­actions by the presence of adjacent red and blue triangles; if there are no adjacent red and/or blue triangles, then there are no π–π inter­actions. Fig. 5 ▸ clearly suggests that there are no π⋯π inter­actions in (I).

**Figure 6:**
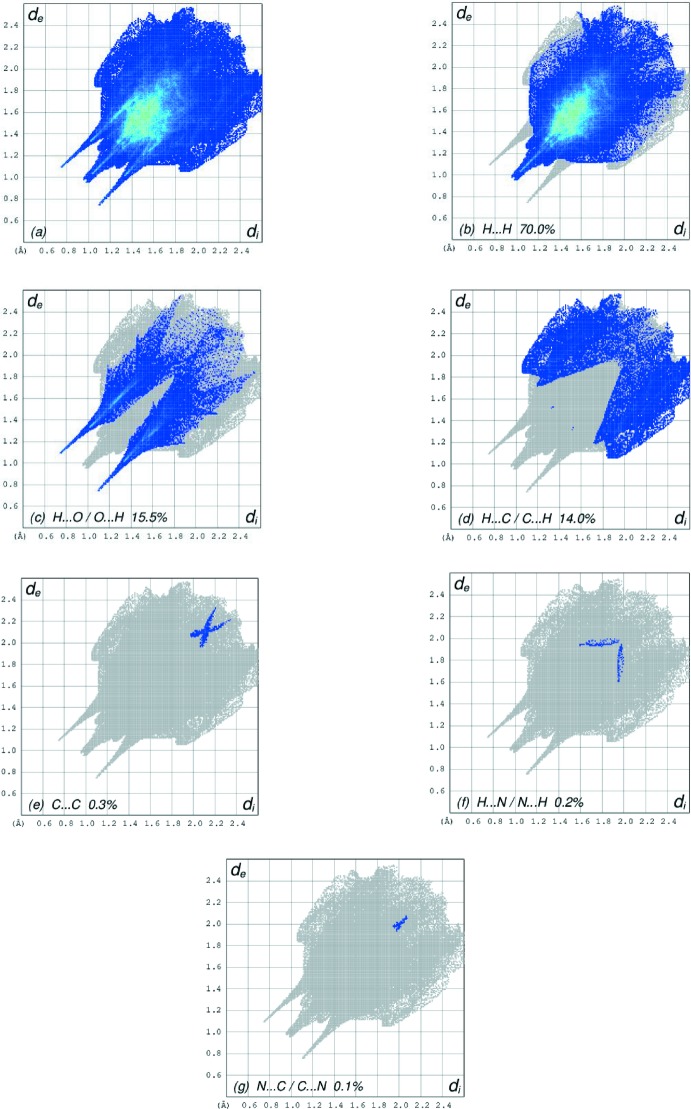
The full two-dimensional fingerprint plots for the title complex, showing (*a*) all inter­actions, and delineated into (*b*) H⋯H, (*c*) H⋯O/O⋯H, (*d*) H⋯C/C⋯H, (*e*) C⋯C, (*f*) H⋯N/N⋯H and (*g*) N⋯C/C⋯N inter­actions. The *d*_i_ and *d*_e_ values are the closest inter­nal and external distances (in Å) from given points on the Hirshfeld surface contacts.

**Figure 7:**
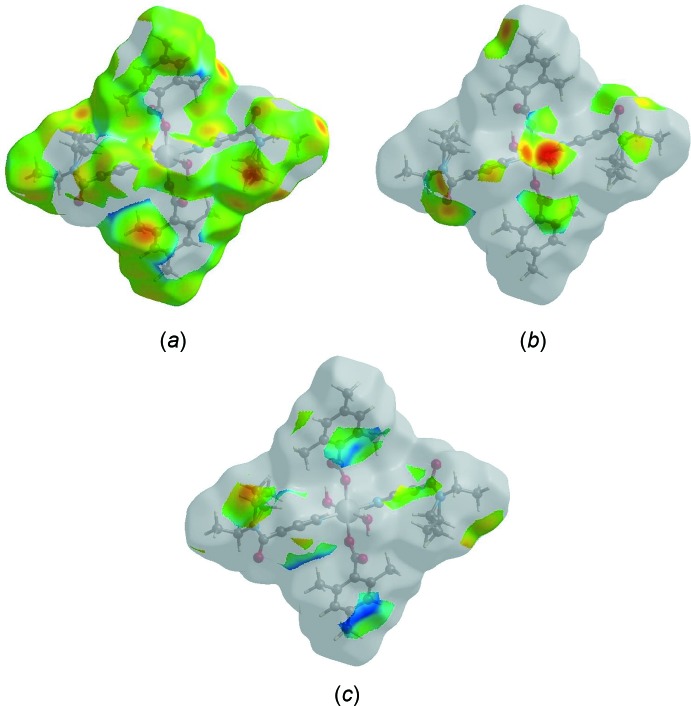
The Hirshfeld surface representations with the function *d*_norm_ plotted onto the surface for (*a*) H⋯H, (*b*) H⋯O/O⋯H and (*c*) H⋯C/C⋯H inter­actions.

The overall two-dimensional fingerprint plot, Fig. 6 ▸ *a*, and those delineated into H⋯H, H⋯O/O⋯H, H⋯C/C⋯H, C⋯C, H⋯N/N⋯H and N⋯C/C⋯N contacts (McKinnon *et al.*, 2007 ▸) are illustrated in Fig. 6 ▸ *b*–*g*, respectively, together with their relative contributions to the Hirshfeld surface. The most important inter­action is H⋯H, contributing 70.0% to the overall crystal packing, which is reflected in Fig. 6 ▸ *b* as widely scattered points of high density due to the large hydrogen content of the mol­ecule. The single spike in the centre at *d*_e_ = *d*_i_ = 0.96 Å in Fig. 6 ▸ *b* is due to a short inter­atomic H⋯H contact (Table 2 ▸). In the fingerprint plot delineated into H⋯O/O⋯H contacts Fig. 6 ▸ *c*, the 15.5% contribution to the HS arises from inter­molecular O—H⋯O hydrogen bonding and is viewed as pair of spikes with the tip at *d*_e_ + *d*_i_ ∼1.84 Å. The short H⋯O/O⋯H contacts may be masked by strong O—H⋯O hydrogen bonding in this plot. In the presence of a weak C—H⋯π inter­action in the crystal, the two pairs of characteristic wings in the fingerprint plot delineated into H⋯C/C⋯H contacts with 14.0% contribution to the HS, Fig. 6 ▸ *d*, and the two pairs of thin and thick edges at *d*_e_ + *d*_i_ ∼2.91 and 2.89 Å, respectively, result from short inter­atomic H⋯C/C⋯H contacts (Table 2 ▸). The Hirshfeld surface representations with the function *d*_norm_ plotted onto the surface are shown for the H⋯H, H⋯O/O⋯H and H⋯C/C⋯H inter­actions in Fig. 7 ▸ *a*–*c*, respectively.

The Hirshfeld surface analysis confirms the importance of H-atom contacts in establishing the packing. The large number of H⋯H, H⋯O/O⋯H and H⋯C/C⋯H inter­actions suggest that van der Waals inter­actions and hydrogen bonding play the major roles in the crystal packing (Hartwar *et al.*, 2015 ▸).

## Synthesis and crystallization   

The title compound was prepared by the reaction of MnSO_4_·H_2_O (0.85 g, 5 mmol) in H_2_O (100 ml) and *N*,*N*-di­ethyl­nicotinamide (1.78 g, 10 mmol) in H_2_O (10 ml) with sodium 2,4,6-tri­methyl­benzoate (1.86 g, 10 mmol) in H_2_O (150 ml). The mixture was filtered and set aside to crystallize at ambient temperature for three weeks, giving colourless single crystals.

## Refinement   

The experimental details including the crystal data, data collection and refinement are summarized in Table 3 ▸. Water H atoms H41 and H42 were located in a difference-Fourier map and freely refined. C-bound H atoms were positioned geometrically, with C—H = 0.93, 0.96 and 0.97 Å for aromatic, methyl and methyl­ene H atoms, respectively, and constrained to ride on their parent atoms, with *U*_iso_(H) = *k* × *U*_eq_(C), where *k* = 1.5 for methyl H atoms and *k* = 1.2 for other H atoms. The disordered ethyl group (C17, C18) was refined over two sets of sites with distance restraints and SIMU and DELU restraints (Sheldrick, 2008 ▸). The refined occupancy ratio of the two orientations is 0.282 (10):0.718 (10).

## Supplementary Material

Crystal structure: contains datablock(s) I, global. DOI: 10.1107/S2056989018003377/xu5920sup1.cif

Structure factors: contains datablock(s) I. DOI: 10.1107/S2056989018003377/xu5920Isup2.hkl

CCDC reference: 1826038

Additional supporting information:  crystallographic information; 3D view; checkCIF report

## Figures and Tables

**Table 1:** Hydrogen-bond geometry (Å, °)

*D*—H⋯*A*	*D*—H	H⋯*A*	*D*⋯*A*	*D*—H⋯*A*
O4—H41⋯O3^i^	0.85 (3)	2.00 (3)	2.838 (2)	171 (3)
O4—H42⋯O2^ii^	0.80 (3)	1.90 (3)	2.660 (3)	157 (3)
C9—H9*C*⋯O2^ix^	0.96	2.48	3.366 (5)	154
C11—H11⋯O3^i^	0.93	2.52	3.447 (3)	179

Symmetry codes: (i) 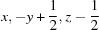; (ii) 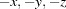; (ix) 
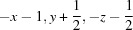
.

**Table 2:** Selected interatomic distances (Å)

O1⋯H10*B*	2.87	C17*A*⋯H15	2.78
O1⋯H13^i^	2.65	C17*B*⋯H20*B*	2.75
O1⋯H8*C*	2.82	C18*A*⋯H9*B* ^v^	2.87
O2⋯H42^ii^	1.90 (3)	C18*B*⋯H8*B* ^vi^	2.79
O2⋯H9*C* ^iii^	2.48	C20⋯H17*C*	2.76
O3⋯H12^iv^	2.85	H4⋯H8*A*	2.37
O3⋯H11^v^	2.52	H4⋯H9*A*	2.38
O3⋯H41^v^	2.00 (3)	H6⋯H10*A*	2.37
O3⋯H19*B*	2.35	H6⋯H9*C*	2.50
O4⋯H15^ii^	2.62	H8*A*⋯H20*A* ^vii^	2.31
O4⋯H11	2.89	H8*B*⋯H17*A* ^viii^	2.44
C1⋯H42^ii^	2.61 (3)	H8*B*⋯H18*E* ^viii^	2.14
C1⋯H8*C*	2.59	H11⋯H41	2.52
C1⋯H10*B*	2.71	H15⋯H18*F*	2.48
C14⋯H17*D*	2.40	H15⋯H17*B*	2.00
C14⋯H17*B*	2.74	H17*A*⋯H19*A*	1.96
C14⋯H18*B*	2.82	H17*C*⋯H20*B*	2.16
C15⋯H17*D*	2.88	H18*A*⋯H9*B* ^v^	2.50
C15⋯H17*B*	2.44	H18*E*⋯H19*A*	2.46
C16⋯H18*B*	2.80		

Symmetry codes: (i) 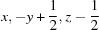; (ii) 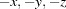; (iii) 
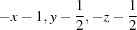
; (iv) 
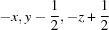
; (v) 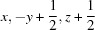; (vi) 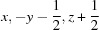; (vii) 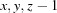; (viii) 
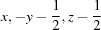
.

**Table 3:** Experimental details

Crystal data	
Chemical formula	[Mn(C_10_H_11_O_2_)_2_(C_10_H_14_N_2_O)_2_(H_2_O)_2_]
*M*_r_	773.83
Crystal system, space group	Monoclinic, *P*2_1_/*c*
Temperature (K)	296
*a*, *b*, *c* (Å)	13.1040 (4), 10.8828 (3), 15.7167 (4)
β (°)	111.570 (2)
*V* (Å^3^)	2084.37 (10)
*Z*	2
Radiation type	Mo *K*α
μ (mm^−1^)	0.37
Crystal size (mm)	0.45 × 0.37 × 0.35

Data collection	
Diffractometer	Bruker *SMART* BREEZE CCD
Absorption correction	Multi-scan (*SADABS*; Bruker, 2012 ▸)
*T*_min_, *T*_max_	0.851, 0.882
No. of measured, independent and observed [*I* > 2σ(*I*)] reflections	36139, 5178, 3995
*R*_int_	0.030
(sin θ/λ)_max_ (Å^−1^)	0.667

Refinement	
*R*[*F* ^2^ > 2σ(*F* ^2^)], *wR*(*F* ^2^), *S*	0.048, 0.144, 1.05
No. of reflections	5178
No. of parameters	274
No. of restraints	42
H-atom treatment	H atoms treated by a mixture of independent and constrained refinement
Δρ_max_, Δρ_min_ (e Å^−3^)	0.51, −0.37

Computer programs: *APEX2* (Bruker, 2012 ▸), *SAINT* (Bruker, 2012 ▸), *SHELXS97* (Sheldrick, 2008 ▸), *SHELXL97* (Sheldrick, 2008 ▸), *ORTEP-3* for Windows (Farrugia, 2012 ▸), *WinGX* (Farrugia, 2012 ▸) and *PLATON* (Spek, 2009 ▸).

## supplementary crystallographic information

### Crystal data

**Table:**

[Mn(C_10_H_11_O_2_)_2_(C_10_H_14_N_2_O)_2_(H_2_O)_2_]	*F*(000) = 822
*M**_r_* = 773.83	*D*_x_ = 1.233 Mg m^−^^3^
Monoclinic, *P*2_1_/*c*	Mo *K*α radiation, λ = 0.71073 Å
Hall symbol: -P 2ybc	Cell parameters from 9985 reflections
*a* = 13.1040 (4) Å	θ = 2.5–28.2°
*b* = 10.8828 (3) Å	µ = 0.37 mm^−^^1^
*c* = 15.7167 (4) Å	*T* = 296 K
β = 111.570 (2)°	Block, translucent light colourless
*V* = 2084.37 (10) Å^3^	0.45 × 0.37 × 0.35 mm
*Z* = 2	

### Data collection

**Table:**

Bruker SMART BREEZE CCD diffractometer	5178 independent reflections
Radiation source: fine-focus sealed tube	3995 reflections with *I* > 2σ(*I*)
Graphite monochromator	*R*_int_ = 0.030
φ and ω scans	θ_max_ = 28.3°, θ_min_ = 1.7°
Absorption correction: multi-scan (*SADABS*; Bruker, 2012)	*h* = −17→17
*T*_min_ = 0.851, *T*_max_ = 0.882	*k* = −14→13
36139 measured reflections	*l* = −20→20

### Refinement

**Table:**

Refinement on *F*^2^	Primary atom site location: structure-invariant direct methods
Least-squares matrix: full	Secondary atom site location: difference Fourier map
*R*[*F*^2^ > 2σ(*F*^2^)] = 0.048	Hydrogen site location: inferred from neighbouring sites
*wR*(*F*^2^) = 0.144	H atoms treated by a mixture of independent and constrained refinement
*S* = 1.05	*w* = 1/[σ^2^(*F*_o_^2^) + (0.0696*P*)^2^ + 0.7599*P*] where *P* = (*F*_o_^2^ + 2*F*_c_^2^)/3
5178 reflections	(Δ/σ)_max_ < 0.001
274 parameters	Δρ_max_ = 0.51 e Å^−^^3^
42 restraints	Δρ_min_ = −0.37 e Å^−^^3^

### Special details

**Table:**

Geometry. All esds (except the esd in the dihedral angle between two l.s. planes) are estimated using the full covariance matrix. The cell esds are taken into account individually in the estimation of esds in distances, angles and torsion angles; correlations between esds in cell parameters are only used when they are defined by crystal symmetry. An approximate (isotropic) treatment of cell esds is used for estimating esds involving l.s. planes.
Refinement. Refinement of F^2^ against ALL reflections. The weighted R-factor wR and goodness of fit S are based on F^2^, conventional R-factors R are based on F, with F set to zero for negative F^2^. The threshold expression of F^2^ > 2sigma(F^2^) is used only for calculating R-factors(gt) etc. and is not relevant to the choice of reflections for refinement. R-factors based on F^2^ are statistically about twice as large as those based on F, and R- factors based on ALL data will be even larger.

### Fractional atomic coordinates and isotropic or equivalent isotropic displacement parameters (Å^2^)

**Table:**

	*x*	*y*	*z*	*U*_iso_*/*U*_eq_	Occ. (<1)
Mn1	0.0000	0.0000	0.0000	0.04559 (14)	
O1	−0.14717 (12)	0.04378 (15)	−0.10765 (9)	0.0615 (4)	
O2	−0.25400 (18)	−0.1155 (2)	−0.11016 (17)	0.1263 (10)	
O3	−0.02706 (15)	0.12536 (14)	0.39218 (9)	0.0699 (4)	
O4	0.08409 (16)	0.14917 (14)	−0.04553 (11)	0.0590 (4)	
H41	0.058 (2)	0.220 (3)	−0.0636 (19)	0.077 (8)*	
H42	0.143 (2)	0.153 (2)	−0.005 (2)	0.075 (9)*	
N1	−0.04496 (14)	0.13195 (15)	0.09755 (10)	0.0534 (4)	
N2	−0.1788 (2)	0.0256 (3)	0.30478 (15)	0.0975 (8)	
C1	−0.23618 (19)	−0.0138 (2)	−0.13796 (16)	0.0667 (6)	
C2	−0.32465 (17)	0.0468 (2)	−0.21779 (15)	0.0633 (5)	
C3	−0.3318 (2)	0.0191 (2)	−0.30595 (17)	0.0696 (6)	
C4	−0.4057 (2)	0.0847 (3)	−0.37831 (17)	0.0805 (7)	
H4	−0.4101	0.0677	−0.4375	0.097*	
C5	−0.4726 (2)	0.1739 (3)	−0.36515 (19)	0.0837 (8)	
C6	−0.4672 (2)	0.1957 (3)	−0.2775 (2)	0.0871 (8)	
H6	−0.5142	0.2536	−0.2680	0.104*	
C7	−0.3934 (2)	0.1337 (3)	−0.20238 (17)	0.0763 (6)	
C8	−0.2595 (3)	−0.0781 (3)	−0.3224 (2)	0.0988 (9)	
H8A	−0.2585	−0.0688	−0.3828	0.148*	
H8B	−0.2875	−0.1579	−0.3166	0.148*	
H8C	−0.1862	−0.0697	−0.2781	0.148*	
C9	−0.5484 (3)	0.2479 (4)	−0.4452 (3)	0.1240 (14)	
H9A	−0.5621	0.2031	−0.5009	0.186*	
H9B	−0.5145	0.3251	−0.4483	0.186*	
H9C	−0.6165	0.2623	−0.4370	0.186*	
C10	−0.3879 (3)	0.1594 (4)	−0.1064 (2)	0.1176 (12)	
H10A	−0.4327	0.2295	−0.1070	0.176*	
H10B	−0.3134	0.1760	−0.0676	0.176*	
H10C	−0.4143	0.0892	−0.0836	0.176*	
C11	−0.0577 (2)	0.2526 (2)	0.08377 (14)	0.0671 (6)	
H11	−0.0501	0.2856	0.0319	0.081*	
C12	−0.0817 (3)	0.3307 (2)	0.14275 (16)	0.0805 (8)	
H12	−0.0887	0.4147	0.1311	0.097*	
C13	−0.0952 (2)	0.2834 (2)	0.21932 (14)	0.0661 (6)	
H13	−0.1108	0.3345	0.2604	0.079*	
C14	−0.08498 (16)	0.15840 (18)	0.23330 (11)	0.0508 (4)	
C15	−0.05883 (18)	0.08718 (18)	0.17157 (12)	0.0532 (4)	
H15	−0.0504	0.0030	0.1822	0.064*	
C16	−0.09574 (19)	0.10153 (18)	0.31679 (12)	0.0580 (5)	
C17A	−0.2372 (10)	−0.0494 (10)	0.2202 (6)	0.085 (4)	0.282 (10)
H17A	−0.2608	−0.1285	0.2348	0.102*	0.282 (10)
H17B	−0.1926	−0.0612	0.1835	0.102*	0.282 (10)
C17B	−0.2820 (5)	0.0332 (6)	0.2173 (4)	0.0939 (18)	0.718 (10)
H17C	−0.3462	0.0479	0.2326	0.113*	0.718 (10)
H17D	−0.2752	0.1001	0.1790	0.113*	0.718 (10)
C18A	−0.3339 (15)	0.036 (2)	0.1740 (14)	0.155 (8)	0.282 (10)
H18A	−0.3582	0.0269	0.1089	0.233*	0.282 (10)
H18B	−0.3114	0.1199	0.1902	0.233*	0.282 (10)
H18C	−0.3929	0.0165	0.1940	0.233*	0.282 (10)
C18B	−0.2932 (6)	−0.0862 (6)	0.1678 (5)	0.142 (3)	0.718 (10)
H18D	−0.3531	−0.0811	0.1100	0.213*	0.718 (10)
H18E	−0.3071	−0.1507	0.2038	0.213*	0.718 (10)
H18F	−0.2266	−0.1034	0.1579	0.213*	0.718 (10)
C19	−0.1838 (4)	−0.0419 (4)	0.3858 (2)	0.1213 (14)	
H19A	−0.2042	−0.1267	0.3691	0.146*	
H19B	−0.1118	−0.0415	0.4342	0.146*	
C20	−0.2624 (5)	0.0130 (5)	0.4194 (3)	0.171 (3)	
H20A	−0.2656	−0.0343	0.4699	0.257*	
H20B	−0.3335	0.0141	0.3713	0.257*	
H20C	−0.2402	0.0955	0.4392	0.257*	

### Atomic displacement parameters (Å^2^)

**Table:**

	*U*^11^	*U*^22^	*U*^33^	*U*^12^	*U*^13^	*U*^23^
Mn1	0.0618 (2)	0.0489 (2)	0.02981 (18)	−0.00067 (16)	0.02117 (16)	0.00058 (13)
O1	0.0652 (9)	0.0672 (8)	0.0465 (7)	−0.0039 (7)	0.0140 (6)	0.0079 (6)
O2	0.0932 (14)	0.1235 (18)	0.1255 (18)	−0.0337 (13)	−0.0030 (13)	0.0690 (15)
O3	0.1131 (12)	0.0614 (8)	0.0348 (7)	−0.0133 (8)	0.0266 (7)	−0.0056 (6)
O4	0.0774 (10)	0.0539 (8)	0.0500 (8)	0.0010 (7)	0.0286 (8)	0.0088 (6)
N1	0.0761 (10)	0.0536 (9)	0.0367 (7)	0.0019 (7)	0.0281 (7)	0.0001 (6)
N2	0.127 (2)	0.1230 (19)	0.0551 (12)	−0.0580 (16)	0.0490 (13)	−0.0147 (12)
C1	0.0634 (12)	0.0778 (15)	0.0558 (12)	−0.0048 (10)	0.0184 (10)	0.0167 (10)
C2	0.0560 (11)	0.0751 (13)	0.0557 (11)	−0.0062 (10)	0.0170 (9)	0.0126 (10)
C3	0.0635 (12)	0.0839 (16)	0.0574 (12)	−0.0026 (11)	0.0178 (10)	0.0092 (10)
C4	0.0739 (15)	0.107 (2)	0.0550 (13)	−0.0008 (14)	0.0166 (11)	0.0126 (12)
C5	0.0642 (14)	0.107 (2)	0.0733 (16)	0.0069 (13)	0.0180 (12)	0.0277 (14)
C6	0.0677 (15)	0.105 (2)	0.0923 (19)	0.0159 (14)	0.0339 (14)	0.0174 (16)
C7	0.0677 (13)	0.0976 (18)	0.0669 (14)	−0.0037 (13)	0.0287 (11)	0.0072 (12)
C8	0.117 (2)	0.098 (2)	0.0807 (19)	0.0161 (18)	0.0365 (17)	0.0007 (16)
C9	0.099 (2)	0.162 (4)	0.100 (2)	0.040 (2)	0.0236 (18)	0.059 (2)
C10	0.123 (3)	0.161 (3)	0.080 (2)	0.015 (2)	0.051 (2)	−0.004 (2)
C11	0.1095 (18)	0.0582 (11)	0.0469 (10)	0.0063 (11)	0.0442 (11)	0.0089 (9)
C12	0.143 (2)	0.0507 (11)	0.0622 (13)	0.0147 (13)	0.0547 (15)	0.0080 (10)
C13	0.1038 (17)	0.0565 (11)	0.0481 (10)	0.0091 (11)	0.0397 (11)	−0.0032 (8)
C14	0.0689 (11)	0.0549 (10)	0.0320 (8)	−0.0057 (8)	0.0224 (8)	−0.0043 (7)
C15	0.0819 (13)	0.0471 (9)	0.0360 (8)	0.0005 (9)	0.0280 (8)	−0.0004 (7)
C16	0.0904 (14)	0.0546 (10)	0.0381 (9)	−0.0074 (10)	0.0344 (9)	−0.0077 (7)
C17A	0.129 (9)	0.068 (6)	0.059 (6)	−0.048 (6)	0.037 (5)	−0.022 (4)
C17B	0.098 (4)	0.109 (4)	0.085 (3)	−0.031 (3)	0.046 (3)	−0.017 (3)
C18A	0.121 (12)	0.197 (19)	0.103 (12)	−0.017 (10)	−0.011 (9)	−0.007 (13)
C18B	0.144 (5)	0.143 (5)	0.142 (5)	−0.059 (4)	0.057 (4)	−0.071 (4)
C19	0.183 (4)	0.124 (3)	0.080 (2)	−0.064 (3)	0.077 (2)	−0.0064 (19)
C20	0.164 (4)	0.283 (7)	0.100 (3)	−0.063 (4)	0.088 (3)	−0.019 (3)

### Geometric parameters (Å, º)

**Table:**

Mn1—O1	2.0999 (14)	C9—H9C	0.9600
Mn1—O1^i^	2.0999 (14)	C10—H10A	0.9600
Mn1—O4	2.2230 (15)	C10—H10B	0.9600
Mn1—O4^i^	2.2230 (15)	C10—H10C	0.9600
Mn1—N1	2.3289 (15)	C11—C12	1.377 (3)
Mn1—N1^i^	2.3289 (15)	C11—H11	0.9300
O1—C1	1.254 (3)	C12—H12	0.9300
O2—C1	1.243 (3)	C13—C12	1.379 (3)
O3—C16	1.224 (3)	C13—H13	0.9300
O4—H41	0.85 (3)	C14—C13	1.376 (3)
O4—H42	0.80 (3)	C14—C15	1.380 (2)
N1—C11	1.331 (3)	C14—C16	1.504 (2)
N1—C15	1.334 (2)	C15—H15	0.9300
N2—C17A	1.506 (9)	C16—N2	1.324 (3)
N2—C17B	1.536 (7)	C17A—C18A	1.526 (17)
N2—C19	1.493 (3)	C17A—H17A	0.9700
C2—C1	1.511 (3)	C17A—H17B	0.9700
C2—C3	1.387 (3)	C17B—C18B	1.493 (8)
C2—C7	1.388 (4)	C17B—H17C	0.9700
C3—C8	1.505 (4)	C17B—H17D	0.9700
C4—C3	1.390 (3)	C18A—H18A	0.9600
C4—C5	1.373 (4)	C18A—H18B	0.9600
C4—H4	0.9300	C18A—H18C	0.9600
C5—C6	1.373 (4)	C18B—H18D	0.9600
C5—C9	1.516 (4)	C18B—H18E	0.9600
C6—H6	0.9300	C18B—H18F	0.9600
C7—C6	1.395 (4)	C19—C20	1.447 (6)
C7—C10	1.510 (4)	C19—H19A	0.9700
C8—H8A	0.9600	C19—H19B	0.9700
C8—H8B	0.9600	C20—H20A	0.9600
C8—H8C	0.9600	C20—H20B	0.9600
C9—H9A	0.9600	C20—H20C	0.9600
C9—H9B	0.9600		
			
O1···H10B	2.87	C16···H18B	2.80
O1···H13^ii^	2.65	C16···H41^v^	2.93 (3)
O1···H8C	2.82	C17A···H15	2.78
O2···H42^i^	1.90 (3)	C17B···H20B	2.75
O2···H9C^iii^	2.48	C18A···H9B^v^	2.87
O3···H12^iv^	2.85	C18B···H8B^vi^	2.79
O3···H11^v^	2.52	C18B···H19A	2.97
O3···H41^v^	2.00 (3)	C19···H18E	2.97
O3···H19B	2.35	C20···H17C	2.76
O4···H15^i^	2.62	H4···H8A	2.37
O4···H11	2.89	H4···H9A	2.38
C1···H10C	2.98	H6···H10A	2.37
C1···H42^i^	2.61 (3)	H6···H9C	2.50
C1···H8C	2.59	H8A···H20A^vii^	2.31
C1···H10B	2.71	H8B···H17A^viii^	2.44
C5···H18B^ii^	2.98	H8B···H18E^viii^	2.14
C13···H17D	2.97	H11···H41	2.52
C14···H17D	2.40	H15···H18F	2.48
C14···H17B	2.74	H15···H17B	2.00
C14···H18B	2.82	H17A···H19A	1.96
C15···H17D	2.88	H17C···H20B	2.16
C15···H17B	2.44	H18A···H9B^v^	2.50
C15···H18F	2.97	H18E···H19A	2.46
			
O1^i^—Mn1—O1	180.00 (7)	H9A—C9—H9B	109.5
O1—Mn1—O4	89.54 (6)	H9A—C9—H9C	109.5
O1^i^—Mn1—O4	90.46 (6)	H9B—C9—H9C	109.5
O1—Mn1—O4^i^	90.46 (6)	C7—C10—H10A	109.5
O1^i^—Mn1—O4^i^	89.54 (6)	C7—C10—H10B	109.5
O1—Mn1—N1	90.62 (6)	C7—C10—H10C	109.5
O1^i^—Mn1—N1	89.38 (6)	H10A—C10—H10B	109.5
O1—Mn1—N1^i^	89.38 (6)	H10A—C10—H10C	109.5
O1^i^—Mn1—N1^i^	90.62 (6)	H10B—C10—H10C	109.5
O4^i^—Mn1—O4	180.00 (9)	N1—C11—C12	123.08 (18)
O4—Mn1—N1	92.12 (6)	N1—C11—H11	118.5
O4^i^—Mn1—N1	87.88 (6)	C12—C11—H11	118.5
O4—Mn1—N1^i^	87.88 (6)	C11—C12—C13	119.4 (2)
O4^i^—Mn1—N1^i^	92.12 (6)	C11—C12—H12	120.3
N1—Mn1—N1^i^	180.00 (7)	C13—C12—H12	120.3
Mn1—O4—H41	126.1 (18)	C12—C13—H13	120.9
Mn1—O4—H42	103 (2)	C14—C13—C12	118.18 (18)
H41—O4—H42	111 (3)	C14—C13—H13	120.9
C1—O1—Mn1	130.03 (14)	C13—C14—C15	118.53 (17)
C11—N1—Mn1	123.24 (12)	C13—C14—C16	120.74 (16)
C11—N1—C15	116.92 (16)	C15—C14—C16	120.64 (17)
C15—N1—Mn1	119.84 (12)	N1—C15—C14	123.83 (18)
C16—N2—C17A	126.0 (4)	N1—C15—H15	118.1
C16—N2—C17B	120.1 (3)	C14—C15—H15	118.1
C16—N2—C19	118.5 (2)	O3—C16—N2	122.95 (19)
C19—N2—C17A	108.6 (4)	O3—C16—C14	119.10 (19)
C19—N2—C17B	119.3 (3)	N2—C16—C14	117.94 (18)
O1—C1—C2	114.81 (19)	N2—C17A—C18A	98.7 (12)
O2—C1—O1	125.5 (2)	N2—C17A—H17A	112.0
O2—C1—C2	119.7 (2)	N2—C17A—H17B	112.0
C3—C2—C1	118.9 (2)	C18A—C17A—H17A	112.0
C3—C2—C7	120.9 (2)	C18A—C17A—H17B	112.0
C7—C2—C1	120.1 (2)	H17A—C17A—H17B	109.7
C2—C3—C4	118.4 (2)	N2—C17B—H17C	110.1
C2—C3—C8	120.6 (2)	N2—C17B—H17D	110.1
C4—C3—C8	121.0 (2)	C18B—C17B—N2	107.8 (6)
C3—C4—H4	118.9	C18B—C17B—H17C	110.1
C5—C4—C3	122.1 (3)	C18B—C17B—H17D	110.1
C5—C4—H4	118.9	H17C—C17B—H17D	108.5
C4—C5—C6	118.2 (2)	C17B—C18B—H18D	109.5
C4—C5—C9	120.7 (3)	C17B—C18B—H18E	109.5
C6—C5—C9	121.1 (3)	C17B—C18B—H18F	109.5
C5—C6—C7	122.0 (3)	H18D—C18B—H18E	109.5
C5—C6—H6	119.0	H18D—C18B—H18F	109.5
C7—C6—H6	119.0	H18E—C18B—H18F	109.5
C2—C7—C6	118.3 (2)	N2—C19—H19A	109.3
C2—C7—C10	120.4 (3)	N2—C19—H19B	109.3
C6—C7—C10	121.3 (3)	C20—C19—N2	111.6 (4)
C3—C8—H8A	109.5	C20—C19—H19A	109.3
C3—C8—H8B	109.5	C20—C19—H19B	109.3
C3—C8—H8C	109.5	H19A—C19—H19B	108.0
H8A—C8—H8B	109.5	C19—C20—H20A	109.5
H8A—C8—H8C	109.5	C19—C20—H20B	109.5
H8B—C8—H8C	109.5	C19—C20—H20C	109.5
C5—C9—H9A	109.5	H20A—C20—H20B	109.5
C5—C9—H9B	109.5	H20A—C20—H20C	109.5
C5—C9—H9C	109.5	H20B—C20—H20C	109.5
			
O4—Mn1—O1—C1	168.4 (2)	C1—C2—C3—C8	5.5 (4)
O4^i^—Mn1—O1—C1	−11.6 (2)	C7—C2—C3—C4	3.1 (4)
N1—Mn1—O1—C1	−99.5 (2)	C7—C2—C3—C8	−178.0 (2)
N1^i^—Mn1—O1—C1	80.5 (2)	C1—C2—C7—C6	174.2 (2)
O1—Mn1—N1—C11	−62.66 (19)	C1—C2—C7—C10	−6.0 (4)
O1^i^—Mn1—N1—C11	117.34 (19)	C3—C2—C7—C6	−2.3 (4)
O1—Mn1—N1—C15	117.53 (16)	C3—C2—C7—C10	177.6 (3)
O1^i^—Mn1—N1—C15	−62.47 (16)	C5—C4—C3—C2	−1.1 (4)
O4—Mn1—N1—C11	26.91 (19)	C5—C4—C3—C8	−179.9 (3)
O4^i^—Mn1—N1—C11	−153.09 (19)	C3—C4—C5—C6	−1.7 (4)
O4—Mn1—N1—C15	−152.91 (16)	C3—C4—C5—C9	177.1 (3)
O4^i^—Mn1—N1—C15	27.09 (16)	C4—C5—C6—C7	2.6 (4)
Mn1—O1—C1—O2	−2.5 (4)	C9—C5—C6—C7	−176.2 (3)
Mn1—O1—C1—C2	−179.51 (14)	C2—C7—C6—C5	−0.6 (4)
Mn1—N1—C11—C12	−178.4 (2)	C10—C7—C6—C5	179.5 (3)
C15—N1—C11—C12	1.4 (4)	N1—C11—C12—C13	−1.1 (4)
Mn1—N1—C15—C14	179.68 (16)	C14—C13—C12—C11	−0.6 (4)
C11—N1—C15—C14	−0.1 (3)	C15—C14—C13—C12	1.7 (4)
C16—N2—C17A—C18A	−96.6 (11)	C16—C14—C13—C12	178.3 (2)
C17B—N2—C17A—C18A	−0.4 (10)	C13—C14—C15—N1	−1.5 (3)
C19—N2—C17A—C18A	113.2 (10)	C16—C14—C15—N1	−178.04 (19)
C16—N2—C17B—C18B	117.7 (4)	C13—C14—C16—O3	−65.7 (3)
C17A—N2—C17B—C18B	6.0 (7)	C13—C14—C16—N2	115.6 (3)
C19—N2—C17B—C18B	−78.9 (5)	C15—C14—C16—O3	110.9 (2)
C16—N2—C19—C20	102.2 (4)	C15—C14—C16—N2	−67.9 (3)
C17A—N2—C19—C20	−105.0 (7)	O3—C16—N2—C17A	−152.8 (7)
C17B—N2—C19—C20	−61.5 (5)	O3—C16—N2—C17B	158.4 (3)
C3—C2—C1—O1	89.8 (3)	O3—C16—N2—C19	−5.2 (4)
C3—C2—C1—O2	−87.4 (3)	C14—C16—N2—C17A	26.0 (8)
C7—C2—C1—O1	−86.7 (3)	C14—C16—N2—C17B	−22.9 (4)
C7—C2—C1—O2	96.0 (3)	C14—C16—N2—C19	173.5 (3)
C1—C2—C3—C4	−173.4 (2)		

Symmetry codes: (i) −*x*, −*y*, −*z*; (ii) *x*, −*y*+1/2, *z*−1/2; (iii) −*x*−1, *y*−1/2, −*z*−1/2; (iv) −*x*, *y*−1/2, −*z*+1/2; (v) *x*, −*y*+1/2, *z*+1/2; (vi) *x*, −*y*−1/2, *z*+1/2; (vii) *x*, *y*, *z*−1; (viii) *x*, −*y*−1/2, *z*−1/2.

### Hydrogen-bond geometry (Å, º)

**Table:**

*D*—H···*A*	*D*—H	H···*A*	*D*···*A*	*D*—H···*A*
O4—H41···O3^ii^	0.85 (3)	2.00 (3)	2.838 (2)	171 (3)
O4—H42···O2^i^	0.80 (3)	1.90 (3)	2.660 (3)	157 (3)
C9—H9*C*···O2^ix^	0.96	2.48	3.366 (5)	154
C11—H11···O3^ii^	0.93	2.52	3.447 (3)	179

Symmetry codes: (i) −*x*, −*y*, −*z*; (ii) *x*, −*y*+1/2, *z*−1/2; (ix) −*x*−1, *y*+1/2, −*z*−1/2.
